# New Insights on Biofilm Antimicrobial Strategies

**DOI:** 10.3390/antibiotics10040407

**Published:** 2021-04-09

**Authors:** Luís D. R. Melo, Nuno F. Azevedo

**Affiliations:** 1 Centre of Biological Engineering, University of Minho, 4710-057 Braga, Portugal; 2 LEPABE—Laboratory for Process Engineering, Environment, Biotechnology and Energy, Department of Chemical Engineering, Faculty of Engineering of the University of Porto, Rua Dr Roberto Frias, 4200-465 Porto, Portugal

Over the last few decades, the study of microbial biofilms has been gaining interest among the scientific community. These microbial communities comprise cells adhered to surfaces that are surrounded by a self-produced exopolymeric matrix that protects biofilm cells against different external stresses. Biofilms can have a negative impact on different sectors within society, namely in agriculture, food industries, and veterinary and human health. As a consequence of their metabolic state and matrix protection, biofilm cells are very difficult to tackle with antibiotics or chemical disinfectants. Due to this problem, recent advances in the development of antibiotic alternatives or complementary strategies to prevent or control biofilms have been reported. This Special Issue includes different strategies to prevent biofilm formation or control biofilm development and includes full research articles, reviews, a communication, and a perspective. 

Regarding the problem per se, Uruén and Chopo-Escuin et al. [1] reviewed the mechanisms by which biofilms are tolerant or resistant to antibiotics, emphasizing the role of the biofilm matrix, physiological heterogeneity of biofilm cells, quorum sensing, horizontal gene transfer, and other mutations on biofilms. In the second part of the review, several alternatives to combat biofilms were discussed. The problem of bacterial resistance was assessed in an original study by Shenkutie et al. [2], where the biofilm-forming ability of 104 *Acinetobacter baumannii* clinical strains was evaluated. Moreover, the authors observed that the minimum biofilm eradication concentrations were significantly higher than the minimum bactericidal concentrations for several antibiotics, a fact that led to an increase in persister cell detection on biofilms. In another study, the influence of *Escherichia coli* diversity in biofilms composed of up to six different strains isolated from urine was evaluated in urinary tract infection conditions. The authors detected that as the number of strains increased, the number of culturable cells also increased but overall the biofilms produced less matrix [3]. The impact of the biofilm matrix on flow cytometry in multi-species biofilms was one of the parameters evaluated by Grainha et al. [4]. Despite the potential of this technique to assess several aspects of biofilms, the authors reported that results are very dependent of the microbial strain used, the morphological state of the cells, and the biofilm matrix.

Another important topic covered in this Special Issue is the prevention of biofilm formation. Alves et al. studied the initial events of *E. coli* adhesion to polydimethylsiloxane and demonstrated that a proper tuning of operational parameters is required to avoid hydrodynamic blocking, which will allow the scientific community to obtain reliable data about cell–surface interactions [5]. In another study performed by the same group, the authors show the effect of pristine and functionalized carbon nanotube incorporation into poly(dimethylsiloxane) materials. Initial *E. coli* adhesion was assessed in conditions simulating urinary tract devices (catheters and stents). The results led the authors to conclude that the incorporation of carbon nanotubes, even at low loading values, might be beneficial for the application of biomedical devices for the urinary tract [6].

Trøstrup et al. and Brum et al. contributed to this Special Issue with two reviews [7,8]. The first is focused on the impact of Pseudomonas aeruginosa biofilms on the local and systemic host response observed in vitro and in vivo. The authors also discussed the implications for clinical wound healing and a possible therapeutic approach using an antimicrobial peptide as immunomodulatory topical treatment [8]. In the second review, a comparison between the use of polyether-ether-ketone (PEEK) and other commonly used materials in implant dentistry (titanium and zirconia) as biofilm-preventing or -controlling agents was comprehensively conducted. The authors concluded that despite pure PEEK being susceptible to biofilm formation, there are numerous strategies that can improve its antibiofilm properties, namely PEEK sulfonation, incorporation of therapeutic/bioactive agents in the PEEK matrix or surface, PEEK coatings, and, finally, the incorporation of reinforcement agents [7].

Different approaches to control biofilms using antibiotic alternative strategies were also submitted to this Special Issue. James D. Boub provided a perspective on the use of phage therapy to combat infectious biofilms [9]. The perspective referred to many aspects of bacteriophage therapy that should be taken into consideration before their broader use. The author suggested the development of standardized protocols that will allow for better and stricter testing of this therapeutic agent in the treatment of biofilm infections. Regarding this topic, Oliveira et al. used a bacteriophage cocktail to control *P. aeruginosa* biofilm formation on endotracheal tubes [10]. Despite some promising results on reducing bacterial colonization, the authors concluded that this strategy could have more potential with the development of new coating strategies. Several natural products are also commonly seen as promising antibiofilm agents. Hoang et al. analyzed the composition and antibiofilm activity of 15 methanolic extracts from *Iris* spp. [11]. *Iris pallida* s.l. leaf extract was the most effective at both preventing biofilm formation and controlling multi-species oral biofilms, with no toxicity observed, suggesting its potential application for oral biofilms. In another study, the antibiofilm activity of cyanobacteria *Arthrospira platensis* extracts (free and nanovectorized) was studied on *Candida albicans* and *Cutibacterium acnes* (single- and dual-species biofilms). Efficacy results varied depending on the microbial species and on the type of biofilm, emphasizing the importance of studying more complex communities such as polymicrobial biofilms [12]. 

Using a different approach, Mil-Homens et al. reported the application of a synthetic polycationic oligomer (L-OEI-h) as an alternative to treat *Klebsiella pneumoniae* infections [13]. The authors showed that L-OEI-h caused lysis of the cytoplasmic membrane in a panel of different species. This promising compound showed no visible cytotoxicity on the *Galleria mellonella* in vivo model; however, its antibiofilm capacity is yet to be tested. 

The last approach published in this Special Issue was the use of electrospun titanium dioxide (TiO_2_) nanofibers and their activity against *Staphylococcus aureus* and *P. aeruginosa* [14]. Although, on planktonic cultures, TiO_2_ nanofibers were more active against *P. aeruginosa* than *S. aureus*, biofilms were prevented in both species in the same order of magnitude, suggesting their potential use for coating inanimate objects.

From a different perspective, Verran et al. provided a communication discussing the relevance of public engagement activities in complex phenomena such as biofilms and Antimicrobial resistance (AMR) [15]. The authors describe three different public engagement activities focused on biofilm control, namely hand hygiene, plaque control, and an externally applied antimicrobial coating using quantitative and/or qualitative methods.

This Special Issue collects high-quality original articles and reviews that demonstrate the relevance of biofilm infections as well as the potential of numerous antibiotic alternative strategies to prevent or control them. We hope that these articles will encourage researchers to investigate new antibiofilm strategies and implement them using standardized protocols that will help research to more effectively move towards clinical implementation. 
